# Quality and readability of web-based Arabic health information on periodontal disease

**DOI:** 10.1186/s12911-021-01413-0

**Published:** 2021-02-04

**Authors:** Mohammed Sultan Al-Ak’hali, Hytham N. Fageeh, Esam Halboub, Mohammed Nasser Alhajj, Zaihan Ariffin

**Affiliations:** 1 Department of Preventive Dental Sciences, College of Dentistry, Jazan University, Jazan, Saudi Arabia; 2 Department of Periodontology, Faculty of Dentistry, Sana’a University, Sana’a, Yemen; 3 Department of Preventive Dental Sciences, College of Dentistry, Jazan University, Jazan, Saudi Arabia; 4 Department of Maxillofacial Surgery and Diagnostic Sciences, College of Dentistry, Jazan University, Jazan, Saudi Arabia; 5 Department of Oral Medicine, Oral Pathology and Oral Radiology, Faculty of Dentistry, Sana’a University, Sana’a, Yemen; 6 Prosthodontics Unit, School of Dental Sciences, Health Campus, Universiti Sains Malaysia, 16150 Kubang Kerian, Kelantan, Malaysia

**Keywords:** Health information, Infodemiology, Misinformation, Periodontal disease, Quality, Readability

## Abstract

**Background:**

Currently, the Internet seems to be a helpful tool for obtaining information about everything that we think about, including diseases, their prevention and treatment approaches. However, doubts exist regarding the quality and readability of such information. This study sought to assess the quality and readability of web-based Arabic information on periodontal disease.

**Methods:**

In this infodemiological study, the Google, Yahoo!, and Bing search engines were searched using specific Arabic terms on periodontal disease. The first 100 consecutive websites from each engine were obtained. The eligible websites were categorized as commercial, health/professional, journalism, and other. The following tools were applied to assess the quality of the information on the included websites: the Health on the Net Foundation Code of Conduct (HONcode), the *Journal of the American Medical Association* (JAMA) benchmarks, and the DISCERN tool. The readability was assessed using an online readability tool.

**Results:**

Of the 300 websites, 89 were eligible for quality and readability analyses. Only two websites (2.3%) were HONcode certified. Based on the DISCERN tool, 43 (48.3%) websites had low scores. The mean score of the JAMA benchmarks was 1.6 ± 1.0, but only 3 (3.4%) websites achieved “yes” responses for all four JAMA criteria. Based on the DISCERN tool, health/professional websites revealed the highest quality of information compared to other website categories. Most of the health/professional websites revealed moderate-quality information, while 55% of the commercial websites, 66% of journalism websites, and 43% of other websites showed poor quality information. Regarding readability, most of the analyzed websites presented simple and readable written content.

**Conclusions:**

Aside from readable content, Arabic health information on the analyzed websites on periodontal disease is below the required level of quality.

## Background

Periodontal disease is a general term describing a chronic infectious destructive condition of the gingiva and the supporting connective tissue and alveolar bone, which together anchor the teeth in the jaws [1]. It is a highly prevalent disease worldwide, ranking as the second burden dental disease after dental caries, with an estimated prevalence rate of 20 to 50% that increases with age [2, 3]. Unless managed properly, periodontitis ultimately ends with tooth loss, which dramatically affects the patient’s function and quality of life [4]. Both professional periodontal treatment and dental home care are equally important for the management of patients with periodontal disease [5–7]. For their part, dental practitioners perform mechanical and/or surgical treatment, along with providing instructions and motivations to their patients. It is the entire responsibility of these patients to strictly follow these instructions and motivations for the greatest benefit.

The increasingly popular and ubiquitous Internet is changing the way in which we access information. Today, almost all patients browse different websites seeking to clarify their health-related concerns, even before consulting specialists. Murray et al. found that 85% of physicians reported a patient bringing Internet information ahead of the planned visit [8]. Overall, clinicians, including periodontists, have faced patients who have been misinformed due to browsing websites searching for health information without professional guidance [9, 10]. Therefore, these health-related websites must be assessed for their quality and regularly supervised since they not only concern health care consumers but also researchers and clinicians [11–13]. Thus, exploring the quality of web-based health data is of paramount importance. Basically, the higher that the quality is of the health information on the Internet, the greater that the benefits and the less that the harm will be. The main challenge is that Arabic studies of web-based information about periodontal diseases have been lacking, along with a scarcity of English studies in this context. Bizzi et al. used JAMA benchmarks to assess web-based health information about periodontal health and found that Google’s first ten results had higher JAMA scores than the remaining websites [14]. Another study unfortunately reported that health information on periodontitis on German dentists’ websites was not fully trustworthy and included irrelevant information [15]. Relying on such unreliable information can lead to non-evidence-based therapy and likely harm [16]. To the best of our knowledge, no single study so far has assessed web-based Arabic health information on periodontal disease. Since up to 51.6% of Arabic-speaking populations use the Internet [17], the assessment of the Arabic Internet content must be undertaken regularly at least in the context of health information. We hypothesized that the web-based Arabic information on periodontal disease are of high quality and readable. Hence, this study sought to assess the quality and readability of web-based Arabic health information regarding periodontal disease and its prevention and treatment.

## Methods

In this infodemiological study, selected search engines (see below) were searched aiming to retrieve information on periodontal disease using relevant Arabic terms.

### Search strategy

Using Google Chrome, version 81.0.4044, we searched the following engines: Google (http://www.google.com), Yahoo! (http://www.yahoo.com), and Bing (http://www.bing.com) on 9 June 2020. This step was performed according to “The Pew Research Center’s Internet & American Life Project” [18], which stated that 79% of online health seekers use these search engines. Ahead of browsing and searching, cookie information was erased. To prevent any potential biases that could arise from preceding searches, we browsed using “incognito” (private) mode.

**Fig. 1:**
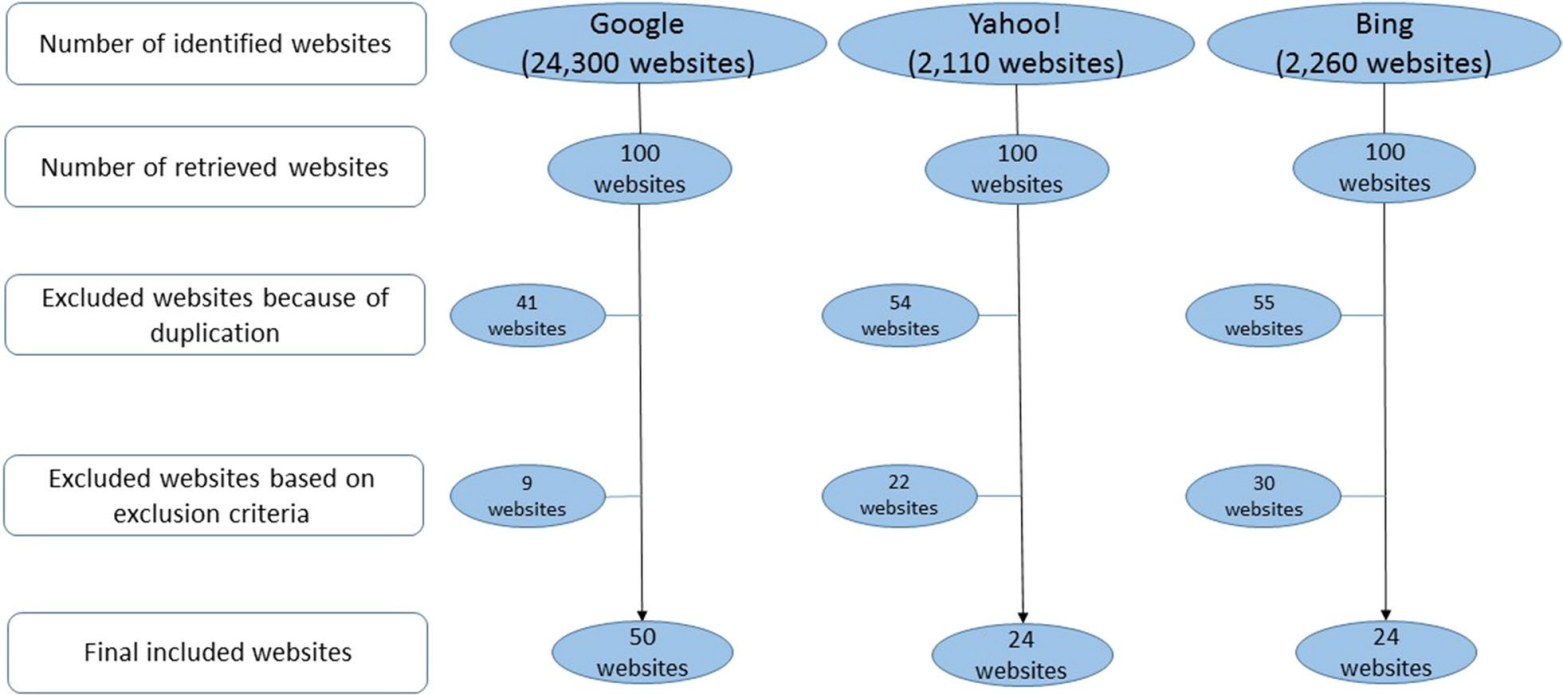
Flow chart of the search strategy

The Arabic translations of the most widely used English terms describing periodontal disease and its treatment were used as search keywords: “gingival inflammation,” “gingivitis,” “gingival disease,” “gingival health,” “periodontal inflammation,” “periodontitis,” “periodontal disease,” “periodontal health,” “periodontal pockets,” “gingival recessions,” and “periodontal treatment.” The first 100 consecutive websites (the first 10 consecutive pages) from each engine were obtained. The duplicates were checked and when present were removed. The relevant websites presenting health information about periodontal disease in the Arabic language were selected for subsequent evaluation. The following criteria were applied to exclude websites: (1) non-Arabic language; (2) information presented only in hints or exclusively audio or video based; (3) complete scientific articles or textbooks; (4) the presence of banner advertisements or sponsored links and discussion forums; (5) blocked sites or sites with denied direct access (required ID and password); and (6) social forums and social media websites. The remaining websites were included for quality and readability analyses. The different stages of the search strategy that we followed are depicted in Fig. 1.

### Quality assessment tools

The following tools were used to assess the quality of the included websites: the Health on the Net Foundation Code of Conduct (HONcode) [19], JAMA benchmarks [20], and the DISCERN tool [21].

The HONcode tool ensures granted permission to display a stamp (HON award-like badge) on a website on the condition that it complies with HONcode criteria. This stamp is a certificate-like badge and remains valid for 1 year only.

Published by the *Journal of the American Medical Association*, the JAMA benchmarks tool evaluates the following points: authorship (availability of data on authors, their contributors, affiliations, and relevant credentials); attribution (availability of clear references and sources from which the content was cited); disclosure (availability of data on ownership, sponsorship, advertising, underwriting, commercial funding or support sources and any potential conflicts of interest); and currency (dates of initial posting and updating of the content were clearly mentioned). Each criterion, when fulfilled (“yes” response) ensured a score of one point for the website; otherwise, it was scored zero (0) points. Hence, the range of the overall JAMA score ranges from 0 (no criteria fulfilled) to 4 points (all 4 criteria fulfilled).

The DISCERN tool includes 16 questions structured into 3 sections: questions 1–8 address the trustfulness of websites as sources of data about selected therapies, questions 9–15 address therapy options, and question 16 measures the overall quality score. Each question is scored from 1 to 5, with 1 indicating a poor website and 5 indicating a good quality website.

Two of the authors (EH and MSA) conducted the quality assessment using the DISCERN and JAMA tools. First, both authors independently assessed 5 websites and resolved any discrepancies, if any, by discussion. Later, interexaminer calibration was calculated for all of the websites. Regarding HONcode, its software was downloaded and incorporated as an extension into Google Chrome. Accordingly, a HONcode seal appeared on the certified websites with each search. For further confirmation, any websites with the HONcode seal were rechecked at the main HONcode website for the currency of its certificate.

For readability, all of the websites were assessed using an online readability calculator tool [22]. This tool was designed primarily to assess English texts. However, it can be used for other languages. This website uses common, well-known analyzing tools to assess the text: Gunning fog index, Coleman Liau index, Flesch Kincaid grade level (FKGL), Automated Readability Index (ARI), Simple Measure of Gobbledygook (SMOG), and Flesch reading ease (FRE). The FRE, FKGL, and SMOG were selected for readability analysis of the Arabic text. The other indices were excluded because their readability analysis formulas are not applicable to Arabic text; unlike English words, Arabic words are comprised of letters linked to each other. The acceptable readability level was set to < 7 for the FKGL and SMOG and ≥ 80.0 for the FRE [23, 24].

### Statistical analysis

All of the statistical analyses were performed using SPSS software, version 21.0 (Statistical Package for Social Sciences [Armonk, NY: IBM Corp.]) Data were presented as frequencies and percentages or means and standard deviations (SDs) as appropriate. For quantitative variables, differences between different website categories were tested using the Kruskal–Willis test followed by Bonferroni’s correction for pairwise comparisons. The potential associations of the qualitative variables with the website categories were tested by the Chi-square test or Fisher’s exact test as appropriate. A *P* value < 0.05 was considered significant.

## Results

The search retrieved a total of 28,670 results from the three engines. Of the 300 screened websites, 150 websites were excluded as duplicates, resulting in 150 websites analyzed for eligibility. Sixty-one websites were excluded due to being not in the Arabic language, presenting irrelevant information, being social forums and presenting audio or video content only. Thus, 89 eligible websites (Additional file 1: Supplementary file) were assessed for quality and readability. Of these 89, only two (2.2%) websites (“msdmanuals.com” and “mayoclinic.org”) were HONcode certified.

Table 1 presents comprehensive descriptive and analytic results of the assessment of the JAMA benchmarks and DISCERN tool. Regarding JAMA benchmarks, the overall mean score for all of the websites was of 1.6 ± 1.0 out of a maximum of 4. The mean score was higher for journalism websites (1.9 ± 0.7) compared to the remaining categories of websites. There were 3 (3.4%) websites achieving all of the JAMA criteria (scoring 4 out of 4). Approximately 15% (n = 14) of websites scored 0 (did not fulfill any of the JAMA criteria), most of which were categorized as “other” (n = 9, 28.1%). Most of the shortcomings per JAMA benchmarks were attributed to non-disclosure of the cited references (attribution); only 14 (15.7%) of all websites did so.

**Table 1:** Quality distribution of Arabic websites according to JAMA benchmarks and DISCERN tool (N = 89)

	ALL (N = 89)	Commercial (N = 20)	Health/Prof (N = 16)	Journalism (N = 21)	Others (N = 32)	*P*
JAMA/Authorship; *Yes responses*	38 (42.7)	3 (15.0)	10 (62.5)	13 (61.9)	12 (37.5)	0.006
JAMA/Attribution; *Yes responses*	14 (15.7)	0 (0.0)	5 (31.3)	2 (9.5)	7 (21.9)	0.043
JAMA/Disclosure; *Yes responses*	58 (65.2)	17 (85.0)	8 (50.0)	18 (85.7)	15 (46.9)	0.003
JAMA/Currency; *Yes responses*	28 (31.5)	5 (25.0)	6 (37.5)	6 (28.6)	11 (34.4)	0.834
JAMA						
No criteria met	14 (15.7)	3 (15.0)	2 (12.5)	0 (0.0)	9 (28.1)	0.132
One criterion met	30 (33.7)	10 (50.0)	5 (31.3)	7 (33.3)	8 (25.0)	
Two criteria met	30 (33.7)	6 (30.0)	5 (31.3)	10 (47.6)	9 (28.1)	
Three criteria met	12 (13.5)	1 (5.0)	2 (12.5)	4 (19.1)	5 (15.6)	
Four criteria met	3 (3.4)	0 (0.0)	2 (12.5)	0 (0.0)	1 (3.1)	
DISCERN						
Low	43 (48.4)	11 (55.0)	4 (25.0)	14 (66.7)	14 (43.8)	0.128
Medium	44 (49.4)	9 (45.0)	11 (68.8)	6 (28.6)	18 (56.2)	
High	2 (2.2)	0 (0.0)	1 (6.2)	1 (4.7)	0 (0.0)	
JAMA (Mean ± SD)	1.6 ± 1.0	1.3 ± 0.8	1.8 ± 1.2	1.9 ± 0.7	1.4 ± 1.2	0.143*
DISCERN (Mean ± SD)	36.4 ± 11.3	32.2 ± 8.2	42.4 ± 13.3	31.9 ± 10.1	39.0 ± 10.9	0.007*ʱ

^*^ Kruskal-Willis test was used

ʱ “Journalism” category was different from other categories based on Bonferroni pairwise comparisons

All other test were done using Chi-square or Fisher exact tests as appropriate

P value is considered significant at < 0.05

Based on the DISCERN tool, only 2 (2.2%) websites revealed a high score, while 43 (48.4%) and 44 (49.4%) websites had low and moderate scores, respectively. Up to 68% of the health/professional websites scored in the moderate range, and only 4 (25%) websites showed low quality. Up to 55% of the commercial sites (trade company and dental centers) and 66% and 43% of websites categorized as “journalism” and “other” (mostly social network sites), respectively, revealed poor quality of information. No single commercial website scored high with the DISCERN tool.

Table 2 presents the readability scores (mean ± SD) of the analyzed websites. The highest mean value on the FRE index was reported for the health/professional websites (99.6 ± 5.8), indicating easy reading. However, no significant statistical differences were found for any of the indices across the different website categories (*P* > 0.05). The mean numbers of words and sentences were reported to be highest for the health/professional websites (1301.3 ± 895.5 and 62.4 ± 41.0, respectively), while they were lowest for the commercial websites (737.1 ± 452.3 and 31.5 ± 19.7, respectively).

**Table 2:** Readability scores (Mean ± SD) of the analyzed website portals (N = 89)

	ALL	Commercial	Health/Prof	Journalism	Others	P*
Number of words	1043.4 ± 668.5	737.1 ± 452.3	1301.3 ± 895.5	1072.6 ± 743.7	1086.7 ± 548.8	0.085
Number of sentences	47.8 ± 35.9	31.5 ± 19.7	62.4 ± 41.0	43.4 ± 35.8	53.6 ± 38.1	0.051
FKGL	6.0 ± 3.7	5.9 ± 3.7	4.9 ± 2.2	7.0 ± 3.9	6.0 ± 4.2	0.370
SMOG	3.2 ± 0.4	3.0 ± 0.1	3.1 ± 0.3	3.2 ± 0.5	3.2 ± 0.4	0.365
FRE	96.7 ± 9.7	96.9 ± 9.7	99.6 ± 5.8	94.1 ± 10.0	96.8 ± 10.9	0.369

^*^ Kruskal-Willis test was used

*P* value is considered significant at < 0.05

More than 60% of websites had FKGL scores less than 7, 100% had a SMOG index less than 7, and more than 90% had reading ease scores equal to or greater than 80. No single website in the health/professional categories reported FRE scores less than 80. More details are presented in Table 3.

**Table 3:** Readability scores of the analyzed website portal based on the acceptable readability levels (N = 89)

	FKGL		SMOG	FRE	
	< 7 score	≥ 7 score	< 7 score	< 80 score	≥ 80 score
ALL	60 (67.4)	29 (33.6)	89 (100.0)	6 (6.7)	83 (93.3)
Commercial	14 (70.0)	6 (30.0)	20 (100.0)	1 (5.0)	19 (95.0)
Health/Prof	11 (68.8)	5 (31.2)	16 (100.0)	0 (0.0)	16 (100.0)
Journalism	14 (66.7)	7 (33.3)	21 (100.0)	3 (14.3)	18 (85.7)
Others	21 (65.6)	11 (34.4)	32 (100.0)	2 (6.3)	30 (93.7)
P*	0.989		NC	0.367	

^*^ Chi-square test was used

A significant, negative, moderate correlation was found between the number of sentences with FKGL (r = − 0.537; *P* < 0.001), while the former was positively correlated with FRE (r = 0.535; *P* < 0.001). Interestingly, a negative, perfect correlation was found between FKGL and FRE (r = − 1.000; *P* < 0.001), indicating more understandable and readable text (Table 4).

**Table 4:** Spearman Correlation Coefficient for between the different readability indices

		FKGL	SMOG	FRE
Number of words	Correlation Coefficient	− 0.142	.245^*^	0.140
	***p***	***0.185***	***0.020***	***0.191***
Number of sentences	Correlation Coefficient	− .537^**^	.225^*^	.535^**^
	***p***	***0.000***	***0.034***	***0.000***
FKGL	Correlation Coefficient		− 0.057	− 1.000^**^
	***p***		***0.598***	***0.000***
SMOG	Correlation Coefficient			0.050
	***p***			***0.640***

^**^. Correlation is significant at the 0.01 level (2-tailed)

^*^. Correlation is significant at the 0.05 level (2-tailed)

## Discussion

It is very different for people to live normally and not encounter the Internet. Although it has revolutionized everything around us and simplified the way in which we obtain information and interact with each other, there remain issues regarding the trustworthiness of this information. This matter applies to all issues, including medical ones; among them is periodontal health, which is a matter of paramount importance owing to the widespread prevalence of periodontal disease and its relation to the teeth, which represent the one's social interface. That is, it is not only because of an exclusive health/sanitary point of view that people care for their periodontal health; it is also for appearance purposes. Hence, people pay special attention to their periodontal/dental health. They might seek treatment of diseased periodontium and preventive measures for apparently healthy periodontium. One essential basis for the treatment and/or prevention of periodontal disease is at-home dental practice. The main information source for these practices is dental practitioners who basically provide instructions and motivations for all dental patients. In the Internet era, however, people seek such information on different websites mostly without professional guidance, and looking for health information is the 3rd most common activity [25, 26]. However, given the enormous amount of information included on the Internet, some of it could mislead readers when it is of poor quality. This assumption is true even for English websites, not to mention the Arabic ones, the health information of which has scarcely been assessed, although in contexts other than periodontal disease. Hence, many tools such as JAMA benchmarks, DISCERN and the HONcode tools were created to evaluate the information on medical sites.

Based on the results of the study, the hypothesis is partially rejected because the quality of the website information on periodontal disease/health is mostly poor, but readable. This finding is in agreement with the results of a recent study on web-based Arabic health information about denture hygiene [27]. Surprisingly, of 89 websites, only two were HONcode certified. The HONcode tool allows web users to judge whether they can trust the information found. Essentially, it was founded as an initiative to ensure high-quality health information. The HONcode certification is requested by web publishers with a self-evaluation step. Then, the HONcode Review Committee conducts a thorough inspection and provides recommendations. Based on compliance with these recommendations, the website is awarded the HONcode seal, which is valid for one year [28].

It was distressing that the mean JAMA score was less than the average (1.6 ± 1.0 out of a maximum of 4). Aside from JAMA addressing different aspects of the websites other than the content (authorship, attribution, disclosure and currency), of which consumers might not be aware, information about these aspects is of paramount importance from the perspective of the scientific community. A website might copy and paste high-quality information from a trusted professional website, but how can consumers trust the site and its content when it does not tell the truth regarding copyrights and other relevant issues? The JAMA benchmark is one of the most streamlined tools for the purpose of quality assessment, allowing the evaluator to quickly discredit websites that lack the most basic components of information transparency and reliability. The most important shortcoming in our study based on JAMA was ascribed to “attribution,” with only 14 (15.7%) of 89 websites mentioning the references that they cited. The second most important shortcoming was related to “currency,” with only 28 sites (31.5%) mentioning when information was posted/updated. It was shocking that only 3 (3.4%) websites met the 4 JAMA criteria.

For its part, the DISCERN tool qualifies the content of health websites using 15 different questions covering different aspects of the included information and one general summarizing question. The overall score ranges from one to five, where 1–2 is considered low quality, 3 moderate quality, and 4–5 high quality. It was surprising that only 2 (2.2%) of the 89 websites scored high on DISCERN tool, while 43 (48.4%) and 44 (49.4%) showed low and moderate quality, respectively. Most of the pitfalls regarding DISCERN tool came from lack of or insufficient information on the mechanisms, risk factors and progression of periodontal disease, essential preventive measures, and the importance of professional (mechanical/surgical) therapy, adjuvant therapy and the alternative therapy, if any.

We classified the websites into four groups: commercial (designed specifically for commercial purposes and product sales or services), journalism (designed for the distribution of news and editorial content), health portal/professional (created by health professionals, experts, or professional organizations and providing access to information or articles on health topics), and other, as many previous studies did [29, 30]. Many of these sites provide a variety of medical information about periodontal disease, traditional periodontal treatments, home tips for periodontal hygiene and using plant herbal medicine information, or what is known as alternative medicine in periodontal therapy. Most of the forums, such as women's or religious forums, and many social websites are of a special nature, and the public is not permitted to access them, so they were excluded. In our study, the websites with higher quality were those classified as “health/professional” (n = 16): the only two HONcode-certified websites, and two of the three websites that met the 4 JAMA benchmarks criteria, were in this category. One of the two websites that scored high with DISCERN tool was health/professional; the other belonged to the “journalism” category. The developers of health/professional websites realize what the transparency, reliability, and validity of health information mean and appreciate it. These websites represent well-known health organization/education/bodies; hence, they work with the highest standards of transparency and professionalism. They greater care about their customers, so they care about the information that they convey.

The websites categorized as “commercial” and “journalism” were found to be of low quality regarding the standard of basic core components of information, including authorship, attribution, disclosure and currency; no single website in these categories fulfilled the 4 criteria of JAMA benchmark, making these websites less transparent and unreliable. Based on the DISCERN tool, up to 55% and 66.7 of the websites in these two categories, respectively, delivered low-quality information, meaning that the content might not be beneficial to consumers and must be addressed cautiously, and they must work on seriously improving. Hence, consumers must be warned about the websites categorized under “commercial,” and “journalism” since the quality of health information therein is questionable. This finding is consistent with the results of a recent study conducted to assess the quality of web-based Arabic information on oral cancer [31].

People can find misleading information while browsing websites. Some of these websites might show wrong information about periodontal disease, some websites copy and paste the content of other websites, which is ethically unacceptable, and some websites might focus only on plant medicine or alternative medicine.

The analysis of the included websites revealed simple and readable text, which is understood by the general public. Moreover, there were significant correlations between the number of sentences with FKGL and FRE scores. That is, with an increased number of sentences, the text becomes more understandable and can be read more easily. Moreover, the relationship between FKGL and FRE was more obvious since all websites with FKGL scores > 7 had the lowest scores for FRE. The website “https://s7tak1.blogspot.com” had the most difficult text content (FKGL score of 20.69 and FRE score of 58.52), perhaps due to long text with many lengthy sentences. In contrast, the website “https://www.madenat-al3ilm.com” had the easiest text content (FKGL score of 0.26 and FRE score of 111.68), mostly owing to the adequate number of short sentences, despite the text being long. Since this study was the first assessing the readability of written content on periodontal disease, it is difficult to compare the results with the available literature. Although our results indicated simply written and easy to read content, this finding is inconsistent with the results on web-based English information on halitosis [32], dental treatment for patients with ischemic heart disease [33], and dental implants [34].

There is an urgent need to improve the quality of web-based Arabic information with regard to periodontal disease. Such websites must publish medical information in accordance with quality standards by displaying the information based on different evaluation tools, and they must continuously compete and become keen to obtain health certificates like HONcode to raise the quality of their content and remain under persistent supervision. Moreover, we are planning to share the high-ranked websites with dental professionals to guide their patients in browsing for information on periodontal disease. Furthermore, upon acceptance of this study, we will share it with different professional and academic bodies to study the shortage of Arabic health information and to undertake the necessary action for improvement.

It is worth noting that there were many limitations to our study. The search was limited to three search engines and included the first one hundred websites from each engine. Furthermore, the quality was assessed using only three tools, although they are the most widely used. Further studies that overcome these limitations are strongly encouraged.

## Conclusions

Almost all of the most easily accessible web-based Arabic health information on periodontal disease does not meet standards for quality, regardless of the level of readability and its ability to be understood by the general population of Arabic speakers. Urgent action must be initiated to monitor the websites that provide Arabic health information on periodontal disease, and dental professionals must emphasize proper guidance and education of their patients to search using trustworthy websites and to avoid low-quality websites.

## Supplementary Information

**Additional file 1:** Websites included in the analysis.

## Data Availability

The dataset supporting the findings of this article is available as a supplementary file.

## Abbreviations

HONcode: the Health on the Net Foundation Code of Conduct
JAMA: Journal of the American Medical Association
FKGL: Flesch Kincaid grade level
ARI: Automated Readability Index
SMOG: Simple Measure of Gobbledygook
FRE: Flesch reading ease
SD: standard deviation
